# Beetle (*Ulomoides dermestoides*) fat improves diabetes: effect on liver and pancreatic architecture and on PPARγ expression

**DOI:** 10.1590/1414-431X20187238

**Published:** 2018-04-06

**Authors:** E.I. Jasso-Villagomez, M. Garcia-Lorenzana, J.C. Almanza-Perez, M.A. Fortis-Barrera, G. Blancas-Flores, R. Roman-Ramos, L.A. Prado-Barragan, F.J. Alarcon-Aguilar

**Affiliations:** 1 Laboratory of Pharmacology, Department of Health Sciences, Division of Health and Biological Sciences, Metropolitan Autonomous University of Iztapalapa, Mexico City, Mexico; 2 Laboratory of Tissue Neurobiology, Department of Reproduction Biology, Division of Health and Biological Sciences, Metropolitan Autonomous University of Iztapalapa, Mexico City, Mexico; 3 Laboratory of Solid State Fermentation, Department of Biotechnology, Division of Health and Biological Sciences, Metropolitan Autonomous University of Iztapalapa, Mexico City, Mexico

**Keywords:** Diabetes, Ulomoides dermestoides, Hypoglycemic agent, Insulin-sensitizing agent, Fatty acids, PPARγ

## Abstract

*Ulomoides dermestoides* is a beetle traditionally consumed to treat diabetes. In this study, we performed a composition analysis of *U. dermestoides* to obtain the principal fractions, which were used to assess the effect on glycemia, liver and pancreatic architecture, and *PPARγ* and *GLUT4* expression. Normal mice and alloxan-induced diabetic mice were administered fractions of chitin, protein or fat, and the acute hypoglycemic effect was evaluated. A subacute study involving daily administration of these fractions to diabetic mice was also performed over 30 days, after which the liver and pancreas were processed by conventional histological techniques and stained with hematoxylin and eosin to evaluate morphological changes. The most active fraction, the fat fraction, was analyzed by gas chromatography-mass spectrometry (GC-MS), and *PPARγ* and *GLUT4* mRNA expressions were determined in 3T3-L1 adipocytes. The protein and fat fractions exhibited hypoglycemic effects in the acute as well as in the 30-day study. Only the fat fraction led to elevated insulin levels and reduced glycemia, as well as lower intake of water and food. In the liver, we observed recovery of close hepatic cords in the central lobule vein following treatment with the fat fraction, while in the pancreas there was an increased density and percentage of islets and number of cells per islet, suggesting cellular regeneration. The GC-MS analysis of fat revealed three fatty acids as the major components. Finally, increased expression of *PPARγ* and *GLUT4* was observed in 3T3-L1 adipocytes, indicating an antidiabetic effect.

## Introduction

Diabetes mellitus is a metabolic disorder characterized by hyperglycemia, caused by a deficit in the secretion or function of insulin (1). Although exogenous insulin and other drugs such as peroxisome proliferator-activated receptor gamma (PPARγ) agonists may help control diabetes (1), long-term patients develop progressive complications including retinopathy, nephropathy, neuropathy, and cardiovascular disease (2). The worldwide cost of diabetes is increasing every year (3). Therefore, the development of new treatments to prevent this illness is important. PPARγ is a member of the nuclear hormone receptor superfamily that regulates glucose and fat metabolism, ameliorates insulin resistance through the mobilization of GLUT4, and increases insulin sensitivity (4).

*Ulomoides dermestoides* Chevrolat, a beetle also known as the “peanut weevil”, is used in the traditional medicine of Argentina, Brazil, China, Colombia, Japan, and Mexico to treat backache, cough, asthma, and diabetes, among others (5,6). The main compounds of the cuticle (alkenes and terpenes) and the defensive secretion (benzoquinones) of this beetle have been characterized in previous chemical studies (7), and its adverse anti-inflammatory, cytotoxic, and genotoxic activities have been described (6–9). Concerning its use in the control of diabetes, it is thought that ingestion of the live adult beetle induces pancreatic regeneration. However, it remains unclear whether *U. dermestoides* has an effect on glycemia, and if it is associated with cellular regeneration at the pancreatic level or with the activation of PPARγ.

In the present study, alloxan-induced diabetic mice were given the principal fractions of *U. dermestoides* (chitin, protein, and fat) to evaluate their effects on glycemia after a single administration (acute study) and after daily administration for 30 days (subacute study) for histological analyses of the liver and pancreas. In addition, with the fat fraction, a gas chromatography-mass spectrometry analysis (CG-MS) was performed and the changes in mRNA expression of PPARγ and GLUT4 were evaluated.

## Material and Methods

### U. dermestoides

*U. dermestoides* beetles were grown to adulthood in a sanitary bed (natural wheat bran) at 25±2°C and fed a diet consisting of banana peels and bread. The taxonomic authentication of the beetle was performed at the Entomology and Acarology Center, Phyto-Sanitary Institute, Postgraduate College of Agricultural Sciences, COLPOS (Mexico).

### Composition analysis of *U. dermestoides*

A sample of *U. dermestoides* was subjected to composition analysis. The moisture, protein, fat, crude fiber, and ash contents were determined according to the methods reported by the Association of Official Analytical Chemists (10). The moisture content was determined in a thermal scale (OHAUS MB45^MR^, Switzerland), and the ash content in a muffle (Thermo Scientific, Germany) at 550°C for 4 h. Total protein content was determined by total digestion (Buchi K-350 Distillation Unit, Switzerland). The ether extraction of crude fat was performed using a Soxhlet extractor (Barlstead™; Thermo Scientific), and crude fiber was quantified using the method described by Hernandez et al. (11).

### Isolation of the fat fraction

Live adult beetles were frozen at −80°C, dehydrated at 60°C and ground to a fine powder. The total fat fraction was obtained from 10 g of dried beetle sample by Soxhlet extraction using petroleum ether as the solvent (12). The fat fraction was recovered in a rotary evaporator (B490; Buchi, Switzerland).

### Isolation of the chitin fraction

The defatted sample (7.6 g) was ground and mixed with 300 mL of 10% NaOH. After incubation at 60°C for 3 h, the slurry was filtered through Whatman no. 4 filter paper. The precipitate (chitin fraction) was dried at 60°C and stored in an airtight container at 2–4°C until use (11). The supernatant was used for soluble protein assays.

### Isolation of the protein fraction

The resultant supernatant from the chitin extraction was subjected to acidification (HCl_conc._) until the isoelectric point of the proteins was reached (pH 3). Then, the sample was filtered through Whatman no. 4 filter paper and the insoluble protein fraction was dried and stored at 2–4°C until use (13).

### Experimental animals

Male *Mus musculus* mice of the CD1 strain (35–45 g) were purchased and bred in the Experimental Animal Center at Metropolitan Autonomous University, Mexico. Six mice per cage were maintained under a 12/12 h light/dark period at 22±1°C and relative humidity of 55±3%. Mice were fed a rodent diet containing 18.6% protein, 44.2% carbohydrates, and 6.2% fat (2018s Teklad Global 18% protein; Harlan Laboratories, USA) and received water *ad libitum*. The handling of the laboratory animals and experimental procedures were performed according to national and international standards including the Official Mexican Standard (NOM-062-ZOO-999, revised 2001) and the National Institutes of Health (NIH publication No. 8023, revised 1996) for the health, safety and comfort of experimental animals. Additionally, the internal committee of the Metropolitan Autonomous University approved the experimental animal handling protocol (DCBS.949.2017).

### Evaluation of the acute hypoglycemic effect of the different fractions of *U. dermestoides* in normal mice

In previous *in vivo* assays performed with *U. dermestoides*, the tested doses of the active extracts ranged from 0.6, 3, 8, and 16 mg/kg (9,14). Therefore, in the present study, only the highest dose (16 mg/kg) was selected to perform all experiments. Thirty experimental animals were fasted for 12 h and grouped as follows: group 1 (control), treated with isotonic saline solution (ISS, 4 mL/kg); group 2, treated with the fat fraction (16 mg/kg); group 3, treated with the protein fraction (16 mg/kg); group 4, treated with the chitin fraction (16 mg/kg); and group 5, positive control, treated with glibenclamide (10 mg/kg), a sulphonyl urea agent with hypoglycemic action that acts at the pancreatic level as an insulin secretagogue. All treatments were administered by the intraperitoneal (*ip*) route and glycemia was quantified from tail vein blood samples at 0, 120, 240, and 360 min by the dehydrogenase method (Accu-Chek™ Performa; Roche Diagnostics, USA).

### Induction of diabetes in mice

Mice were intravenously administrated a single dose of 70 mg/kg of alloxan (Sigma-Aldrich, USA) dissolved in ISS (15). The control group was treated with ISS only. After 8 days of drug administration, glycemia was quantified. Only those alloxan-treated mice with blood glucose levels higher than 200 mg/dL were included in the acute and subacute studies.

### Evaluation of the acute hypoglycemic effect of *U. dermestoides* in alloxan-induced diabetic mice

Thirty mice with alloxan-induced diabetes were grouped as follows: group 1, control, treated with ISS (4 mL/kg); group 2, treated with the lipid fraction (16 mg/kg); group 3, treated with the protein fraction (16 mg/kg); group 4, treated with the chitin fraction (16 mg/kg); and group 5, treated with glibenclamide (10 mg/kg). All treatments were administered *ip* and glycemia was quantified for each group at 0, 120, 240, and 360 min, following the methodology described above for normal mice.

### Evaluation of the subacute hypoglycemic effect of *U. dermestoides*

Six normal mice and 30 mice with experimental diabetes were separated into groups as follows: group 1, normal control, treated with ISS (4 mL/kg); group 2, diabetic control, treated with ISS (4 mL/kg); group 3, diabetic treated with the lipid fraction (16 mg/kg); group 4, diabetic treated with the protein fraction (16 mg/kg); group 5, diabetic treated with the chitin fraction (16 mg/kg); group 6, diabetic treated with glibenclamide (5 mg/kg). Treatments were administered daily by gavage to all groups over a 30-day period, with water and food intake recorded every 24 h. Blood samples (32 μL) were taken from the tail vein by puncture, and cholesterol, triglyceride, aspartate aminotransferase (AST), and alanine aminotransferase (ALT) levels were quantified after 30 days of treatment using a Reflotron Plus System (Bayer, Germany). Glycemia was also quantified after 30 days of treatment by the dehydrogenase method (Accu-Chek™ Performa, Roche Diagnostics).

### Insulin quantification

After 30 days of treatment, mice were anesthetized with sodium pentobarbital (27.5 mg/kg) and blood samples were obtained from the eye orbital sinus. Insulin was quantified by the ELISA method according to the supplier’s instructions (Linco Research, USA).

### Histological analysis

At the end of subacute study (30 days), the animals were anesthetized with sodium pentobarbital (27.5 mg/kg IP) and euthanized according to the Official Mexican Standard (NOM-033-ZOO-1995). The pancreas and liver were obtained surgically and fixed by immersion in 4% paraformaldehyde in phosphate buffer, pH 7.2–7.4, for 18 to 24 h. The organs were processed by a conventional histological technique (16) and embedded in Paraplast™ (Oxford Lab, USA). Serial longitudinal sections of 5-µm thickness were cut on a microtome (Leika Jung Histocut 820, Germany) and stained with hematoxylin and eosin (17). Qualitative analysis was carried out with an optical light microscope (Axioskope II; Carl Zeiss, Germany) coupled to an image analyzer (AxioVision 4.5; Carl Zeiss). Photomicrographs were taken with AxioCamMRc5 (Carl Zeiss).

A morphometric analysis of the islets was performed on longitudinal sections of the pancreas over a total mean area of 387 mm^2^. Analysis of the pancreas was performed on nine serial 5-µm thick sections from three animals in each treatment group. Serial cuts (200) were made over a 150-µm thick area. For this analysis, the sections chosen were those numbered 50, 100, and 200 for each animal. Islet density was determined at 100× magnification, and the islet area and the number of cells per islet were determined at 400× magnification. In total, nine sections per treatment were analyzed by optical light microscopy (Carl Zeiss).

### Gas chromatography-mass spectrometry (GC-MS) analysis

The fat extracted from *U. dermestoides* was analyzed on an Agilent Technologies gas chromatograph (GC) 6890N (USA) coupled to a 5973N mass spectrometer (MS) operated at 70 eV and equipped with a Carbowax capillary column (30 m, 0.25 mm internal diameter) with a polyethylene glycol matrix (0.25 µm thick). Helium was the carrier gas at a flow rate of 1 mL/min. The injector and MS transfer line temperature was set at 250°C. Samples were diluted 1:10 v/v in acetone and manually injected (1.0 µL in volume). After sample injection, the initial temperature of the oven (45°C) was held constant for 3 min and then increased to 135°C at a rate of 3°C/min. This temperature was maintained for 1 min and then increased to 250°C at a rate of 10°C/min, which was maintained for 10 min. For GC-MS detection, an electron ionization system with an ionization energy of 70 eV was used. After a delay of 3 min to allow passage of the solvent, the mass spectra were scanned from 15 to 800 m/z. Tentative identification of the components was performed based on their relative retention time and mass spectra comparison with the National Institute of Standards and Technology Library (NIST, NIST02L) data for the GC-MS system. Quantification of the identified components was based on the integrated peak area.

### 3T3-L1 cell line

The 3T3-L1 fibroblasts were cultured in 6-well plates (Corning Inc., USA) in Dulbecco’s modified Eagle’s medium supplemented with 25 mM glucose, 10% fetal bovine serum (v/v), 1 mM sodium pyruvate, 2 mM glutamine, 0.1 mM nonessential amino acids, and gentamicin in a 5% CO_2_ humidified (95%) atmosphere at 37°C. After cells had reached confluence at 48 h, the cells were treated with 0.5 mM 3-isobutyl-1-methylxanthine, 0.25 µM dexamethasone acetate, and 0.8 µM insulin for 48 h to induce differentiation into an adipocyte phenotype, then 0.8 µM insulin was added and cells were left for a further 48 h. The culture medium without insulin was changed every 2 days over the 8-day cell differentiation period (18).

### MTT assay in 3T3-L1 fibroblasts treated with the fat fraction

Cellular functionality was determined using 3-(4,5-dimethylthiazole-2-yl)-2,5-diphenyltetrazolium bromide (MTT; Sigma, USA) according to the method described by Mosmann (19). The test quantifies the conversion of MTT to formazan by insoluble dehydrogenase enzyme activity of intact cells. 3T3-L1 cells were seeded onto 96-well microplates at a semi-confluent density (5000 cells/well). After 24 h, the medium was replaced with medium containing 1, 10, or 100 μg/mL of fat. Cells were treated for 30 min then washed with phosphate-buffered saline (PBS), pH 7.4, and a solution of MTT at 0.1 mg/mL in PBS, pH 7.4, was added. The cells were incubated for 3 h at 37°C then washed with PBS. Then, 40 mM HCl (200 μL) prepared in isopropanol was added to each well for 15 min to solubilize the produced formazan. Absorbance was read at 570 nm. Data are reported as the percentage of viable cells after treatment with the lipid fraction of *U. dermestoides* compared to control cells.

### Evaluation of mRNA expression of *PPAR-**γ*** and *GLUT4* by RT-PCR in 3T3-L1 adipocytes treated with the fat fraction

Fibroblasts (9×10^5^ cells per well) were cultured and differentiated into adipocytes in 6-well plates (Corning Inc.). The RNA was isolated from cultured cells using a TriPure isolation reagent (Invitrogen, UK). The absorbance was measured at 260 and 280 nm for each RNA sample (the absorbance ratio was 1.9±0.2). To confirm RNA integrity, 1.5 µg was run in a 1% agarose gel and the RNA bands were stained with ethidium bromide and visualized using an Image Gel-Logic 212 Pro (Kodak/Carestream™, USA). Two major ribosomal bands (28S and 18S rRNA) were detected, with no degraded RNA (data not shown).

Total RNA (2 µg) was reverse-transcribed using the ImProm II Reverse Transcription System (Promega, USA), then the mixture (20 µL) was incubated in a thermocycler (Select Cycler; BioProducts, USA), following the cycle program of incubation at 25°C for 5 min and extension at 42°C for 55 min. The enzyme was inactivated at 70°C for 15 min, then samples were cooled to 4°C for 5 min. Then, 1/10 volume of each cDNA sample was amplified with SYBR Green master mix (Roche Molecular Biochemicals, Germany) containing 0.5 mM of customized primers for PPAR-γ (forward 5′-CCAGAGTCTGCTGATCTGCG-3′, reverse 5′-GCCACCTCTTTGCTCTGCTC-3′; Gene Bank NM_011146.1), GLUT4 (forward 5′-GATTCTGCTGCCCTTCTGTC-3′, reverse 5′-ATTGGACGCTCTCTCTCCAA-3′; Gene Bank NM_009204.2), fast-start enzyme, PCR buffer, and 3.5 mM MgCl_2_, made up to a final volume of 10 µL. The reactions were measured by Rotor-Gene Real-Time equipment (Corbett Life Science, Australia).

PCR was conducted using the following cycling conditions: pre-incubation and denaturation at 95°C for 10 min, alignment at 61°C for 7 s, and amplification at 72°C for 10 s. The threshold cycles (Ct) were measured in separate tubes and in duplicate. The identity and purity of the amplified products were checked by electrophoresis on a 2% agarose gel. The melting curve was analyzed at the end of amplification following the SYBR Green kit protocol, as indicated by the company (Roche Molecular Biochemicals). To ensure the quality of the measurements, each assay included a negative control for each gene. The amount of mRNA for each studied parameter was normalized to mRNA encoding the ribosomal protein 36B4 (forward 5′-AAGCGCGTCCTGGCATTGTCT-3′, reverse 5′-CCGCAGGGGCAGCAGTGGT-3′; Gene Bank NM_007475.2). The relative changes in the expression levels of mRNA were calculated with the formula 2^−ΔΔCt^ (20).

### Statistical analysis

Biological data are reported as means±SE. Statistical analysis of the data was performed by two-way analysis of variance (ANOVA) followed by a Tukey-Kramer *post hoc* test at a confidence level of 95% (NCSS 2000, USA).

## Results

### Composition of *U. dermestoides*

The compositional analysis of the beetles based on dried weight revealed the highest percentage was of protein (53.3±1.2%) and fat (24.7±0.7%), followed by fiber (22.1±1.1%), chitin (19.2±0.5%), and ash (1.0±0.3%). In fresh organisms, the moisture content was 56.3±0.3%. The protein, fat, and chitin fractions were selected to be evaluated for their acute hypoglycemic effect in normal and diabetic mice.

### Acute hypoglycemic effect of the fractions

**Figure 1.**
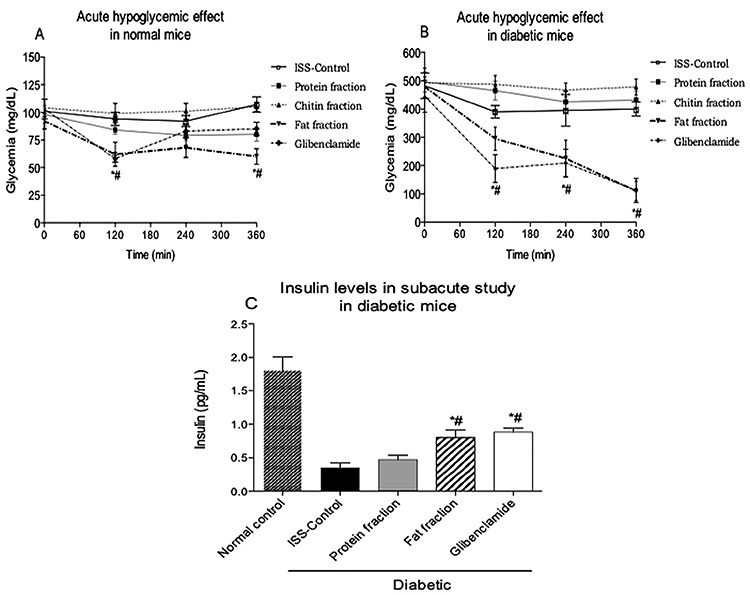
Acute hypoglycemic effect in normal mice (*A*). Acute hypoglycemic effect in alloxan-induced diabetic mice (*B*). Serum insulin levels in the subacute study in normal and alloxan-induced diabetic mice over 30 days (*C*). Mice were treated with either saline (control), protein, chitin or fat fractions (lipid) isolated from *Ulomoides dermestoides*, or glibenclamide. Data are reported as means±SE (n=6). *P<0.05 compared to basal glycemia (or insulin normal control). ^#^P<0.05 compared to isotonic saline solution (ISS)-treated control (ANOVA and Tukey-Kramer *post hoc* test).

The hypoglycemic effects of a single administration of protein, fat or chitin to normal mice are shown in Figure 1A. Only the fat and glibenclamide treatments caused a significant reduction in glycemia at 120 and 360 min compared to the initial glycemia values. Evaluation of the hypoglycemic effect of the components of *U. dermestoides* in alloxan-induced diabetic mice revealed that the fat and glibenclamide treatments caused a significant reduction in glycemia when compared to both the basal glycemia level and to the diabetic control group treated with ISS (Figure 1B). The protein and chitin treatments did not show any hypoglycemic effects in normal or diabetic mice.

### Hypoglycemic effect from daily administration of the fractions

In the subacute study (Figure 1C), administration of the fat fraction was found to increase insulin secretion compared to the diabetic control that received ISS, correcting glycemia as well as water and food intake, with reductions of 80, 65.5, and 58.5%, respectively. No significant difference (P>0.05) in total cholesterol was observed in mice administered this fraction; however, triglyceride levels increased by 50%. Consumption of the protein fraction also caused a significant reduction in glycemia, but there were no significant differences in the group that received the protein fraction compared with the normal control group (Table 1). Finally, chitin treatment (16 mg·kg^−1^·day^−1^) was found to cause 100% lethality after 15 days of treatment. The body weight did not change significantly in any of the treated groups compared with the initial body weight (data not shown).

**Table 1.** Effect of the daily administration for 30 days of the chitin, lipid, and protein fractions from *U. dermestoides* on water intake, food intake, and biochemical parameters.

Parameter	Glycemia (mg/dL)	Water intake (mL/mice)	Food intake (g/mice)	Cholesterol (mg/dL)	Triglycerides (mg/dL)	ALT (U/L)	AST (U/L)
Normal	135±5.5	8.2±0.6	7.2±1.4	100±4.2	70±3.8	8.4±3.8	5.9±1.6
Diabetic (ISS)	593±2.1*	53.5±4.3*	16.7±3.3*	105±3.8	71±9.7	7.2±3.8	198.3±3.3*
Chitin	598±7.8*	59.4±2.9*	17.6±4.6*	–	–	–	–
Fat	120±6.1^#^	18.5±4.6*^#^	6.9±2.4^#^	108±6.8	142±2.9*^#^	6.8±2.1	15.0±3.3*
Proteins	386±16.4*^#^	31.3±2.7*	9.6±0.7	110±7.4	82±6.4	59.1±3.8*^#^	22.5±4.5*^#^
Glibenclamide	552±8.1*	50.2±8.7*	14.2±1.9*	110±5.2	78±6.0	5.9±9.8	23.8±4.8

Data are reported as means±SE (n=6). Chitin values correspond to day 15 of treatment; after this, a 100% lethality was observed. ALT: alanine aminotransferase; AST: aspartate aminotransferase; ISS: isotonic saline solution. *P<0.05 *vs* normal control; ^#^P<0.05 *vs* diabetic control (ANOVA and Tukey-Kramer *post hoc* test).

### Histological changes in the liver and pancreas

**Figure 2.**
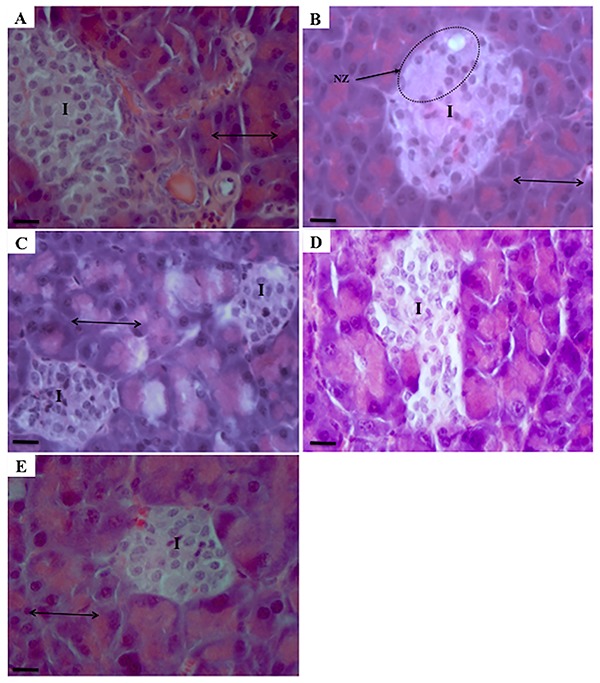
Photomicrographs of pancreatic islets of Langerhans from normal control mice treated with saline (*A*), and diabetic mice treated with saline (diabetic control; *B*), protein fraction (*C*), fat fraction (*D*), or glibenclamide (*E*) for 30 days. The exocrine area (↔), pancreatic islets (I) and the reduced cell number zone (NZ) are indicated. An evident reduction in the islet area and islet cell number can be observed in the saline diabetic control (*B*), while treatment with fat extracted from *Ulomoides dermerstoid*es exhibited normal cell density in the islets (*D*). Slides were stained with hematoxylin and eosin. Magnification: 400×; scale bar: 20 μm.

Micrographs of longitudinal pancreas sections from mice in the different treatment groups are shown in Figure 2. The general structure of the pancreas can be clearly observed in normal mice (Figure 2A), with an exocrine zone consisting of epithelial pancreatic acinus and an endocrine zone of pancreatic islets. Histological analysis showed qualitative and quantitative changes in the typical pancreas architecture after alloxan treatment, particularly in the islets of Langerhans (Figure 2B), with this group displaying necrotic changes in the pancreatic islets. In addition, nuclear changes, karyolysis, and a reduction in the number of islet cells were observed (reduced number of cells per zone).

**Figure 3.**
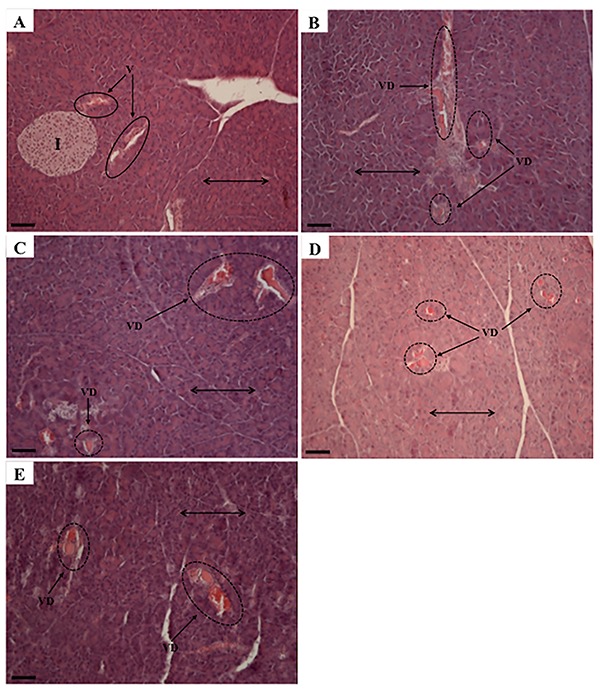
Photomicrographs of longitudinal pancreas sections of normal control mice treated with saline (*A*), and diabetic mice treated with saline (diabetic control; *B*), protein fraction (*C*), fat fraction (*D*) or glibenclamide (*E*) for 30 days. Blood vessels (V), pancreatic islets (I), exocrine area (↔), and blood vessel dilatation (VD) are indicated. The experimental groups showed blood vessel dilatation with hyperemia (B-D). Slides were stained with hematoxylin and eosin. Magnification: 100×; scale bar: 100 μm.

In Figure 2C, pyknotic nuclei are evident in animals treated with the protein fraction of *U. dermestoides*. It is remarkable that with the fat fraction, recovery of the alloxan-induced alterations in pancreatic architecture was observed (Figure 2D). A small number of pyknotic nuclei, improved karyolysis, and a reduction in necrosis suggestive of progression to normal pancreatic morphology were evident. Pancreas samples from mice in the glibenclamide group also showed pyknotic cells, in addition to similar damage to the islets caused by alloxan and regeneration of the central islet region (Figure 2E). Remarkably, the fat fraction increased the number of islets compared to the ISS-treated diabetic control group (41.8% *vs* 11.5%), as well as the number of cells in the islets and the density of the islets (Table 2). In Figure 3, it is evident that all diabetic groups with or without treatment (Figure 3B-E) exhibited hyperemia.

**Table 2.** Histomorphometric analysis of pancreas of alloxan-induced diabetic mice administered isotonic saline solution (ISS) (diabetic control), proteins and fat from *U. dermestoides*, and glibenclamide for 30 days.

Treatment	Islets density (islets/387212.92 mm^2^)	Percent of islets	Islet area (mm^2^)	Number of cells/islet
Normal control	10±2.0	100±1.2	10891.3±164.5	55±8
ISS Diabetic	2±0.7*	11.5±4.7*	10137.3±250.0	15±3*
Proteins	2±0.8*	11.5±4.7*	5977.2±424.5* #	12±2*
Fat	6±1.5*	41.8±3.2* #	12320.2±2820.5	31±3* #
Glibenclamide	1±0.6*	9.8±3.3*	8500.9±1363.5*	14±2*

Data are reported as means±SE (n=6). ISS: isotonic saline solution.

* P<0.05 *vs* normal control;

# P<0.05 *vs* diabetic control (ANOVA and Tukey-Kramer *post hoc* test).

**Figure 4.**
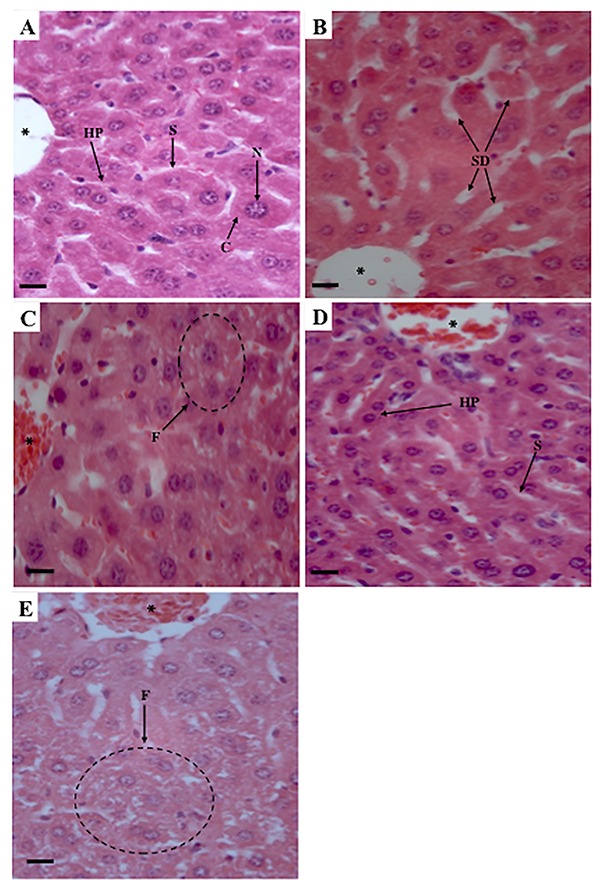
Photomicrographs of longitudinal liver sections of normal control mice treated with saline (*A*), and diabetic mice treated with saline (diabetic control; *B*), protein fraction (*C*), fat fraction (*D*) or glibenclamide (*E*) for 30 days. Lobule central vein (*), hepatocyte plates (HP), sinusoids (S), nucleus (N), cytoplasm (C), sinusoidal dilatation (SD), and fibrosis (F) are indicated. A reduction in sinusoidal dilatation was observed in groups treated with the fat of *Ulomoides dermestoides* (D *vs* B). Slides were stained with hematoxylin and eosin. Magnification: 400×; scale bar: 20 µm.

In the liver histological analysis (Figure 4), hepatocytes of normal mice showed a well-defined nucleus and cytoplasm with cell margins, and the lobule central vein, hepatocytes plates, and the sinusoidal spaces can be clearly observed (Figure 4A). The liver slices from control diabetic mice showed sinusoidal dilatation, a loss of cytoplasmic integrity with indistinct cell margins, and a loss of nuclear staining intensity, nuclear function, and cell death (Figure 4B). The group treated with the protein fraction showed impaired nucleus architecture and mild liver steatosis (Figure 4C). Treatment with the fat fraction showed recovery of the close hepatic cords in the central lobule vein, a well-defined nucleus and cytoplasm, and apparent recovery of cellular function (Figure 4D). Glibenclamide treatment caused changes in the hepatic lobule, including loss of hepatic cords and fibrotic zones (Figure 4E).

### GC-MS analysis of the fat from *U. dermestoides*

The GC-MS analysis of the fat fraction of *U. dermestoides* (Table 3) revealed the presence of 31 different fatty acids (FA), representing 91% of the total components in the fat. The major FA fraction identified was a mixture of linoleic and oleic acids (40.9%) followed by hexadecanoic (palmitic) (31.9%) and octadecanoic (9.3%) acids.

**Table 3.** Composition of the fat from *U. dermestoides*.

Retention time	%	Identified compound
3.702	0.012	Undecane
3.984	0.004	Octanoic acid
5.973	0.008	Decanoic acid
7.678	0.051	Dodecanoic acid
9.221	1.556	Tetradecanoic acid
9.889	0.221	Pentadecanoic acid
10.326	0.151	9-12-hexadecadienoic acid
10.420	1.255	9-hexadecanoic acid
10.806	31.910	Hexadecanoic acid
11.235	0.395	Heptadecanoic acid
11.972	40.957	Linoleic and Oleic acid
12.057	9.280	Octadecanoic acid
12.289	0.062	9-nonadecanoic acid
12.443	0.112	Nonadecanoic acid
12.657	0.073	7,10,13-eicosatrienoic acid
12.794	0.323	11.14-eicosenoic acid
12.837	0.637	11-eicosenoicacid
12.991	0.718	Eocisanoic acid
13.343	0.064	Tetracosane
13.891	1.002	Pentacosane
14.037	0.443	Docosanoic acid
14.868	0.718	Heptacosane
14.997	0.078	Tetracosanoic acid
15.314	0.051	Octacosane
15.597	0.099	Cholesta-3,5-diene
15.777	0.457	Nonacosane
15.914	0.021	Hexacosanoic acid
16.642	0.248	Hentriacontane
16.779	0.054	Octacosanoic acid
17.448	0.192	Tritriacontane
18.416	0.114	Pentatriacontane
	8.735	Unidentified

### Effect of the fat fraction from *U. dermestoides* on *PPARγ* and *GLUT4* expression

**Figure 5.**
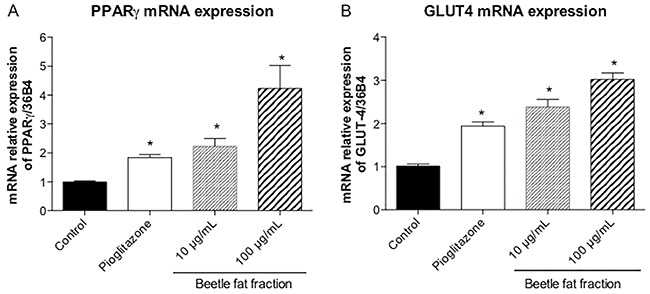
*PPARγ* (*A*) and *GLUT4* (*B*) mRNA expression in 3T3-L1 adipocytes treated with the fat fraction from *Ulomoides dermestoides* and pioglitazone. Data are reported as means±SE (n=6). *P<0.05 compared to control (ANOVA and Tukey-Kramer *post hoc* test).

*In vitro* studies in 3T3-L1 adipocytes performed in our laboratory showed that cell viability was unaffected by treatment with *U. dermestoides* fat at different concentrations (data not shown). Therefore, concentrations of 10 and 100 μg/mL were selected for this experiment. As shown in Figure 5A and B, 100 µg/mL of fat increased the mRNA expression of *PPARγ* (75%) and *GLUT4* (66.6%) compared with the control, and these effects were larger than those observed with pioglitazone, a molecule with PPARγ-agonist activity.

## Discussion

Protein, fat, and chitin were found to be the most abundant chemical components in adult *U. dermestoides* beetles. The protein and lipid fractions had a hypoglycemic effect in the acute experiment; however, in the subacute experiment, only the fat fraction demonstrated a hypoglycemic effect, with an increase in serum insulin as well as a significant reduction in the consumption of food and water. The cholesterol and liver transaminase (ALT and AST) levels did not change, but an increase in triglycerides was observed when mice were supplemented with the fat fraction.

The GC-MS analysis of the fat fraction suggested the presence of 31 different fatty acids, the major components being hexadecanoic (palmitic) acid and a mixture of linoleic and oleic fatty acids, which have been previously reported to be present in methanol and hexane extracts of *U. dermestoides* (21). It is known that consumption of saturated fatty acids, as in the case of hexadecanoic acid, is associated with obesity, inflammation, and insulin resistance (22). In contrast, the consumption of diets high in polyunsaturated and/or monounsaturated fatty acids are considered beneficial for the prevention of various diseases (23). Among the polyunsaturated fatty acids (PUFA), the two most often reported to confer benefits to human and animal health are linoleic and oleic acids, both of which have been associated with anti-diabetic, anti-atherosclerotic, and anti-obesity effects as well as with immune-modulator properties (24). In addition, these fatty acids can act as ligands in transcription factors, functioning as metabolic regulators (25). Nevertheless, it is necessary to make trimethylsylyl derivatives of the fat fraction, as there are likely a number of less volatile derivates contained in the fat fraction that would be observed whether the trimethylsilyl or tert-butyldimethylsilyl derivatives are analyzed (26). This should be further explored in additional studies.

Fatty acids represent an important source of metabolic energy, and can function as physiological signaling molecules. Some fatty acids act as ligands for the peroxisome proliferator-activated receptors (PPAR), especially PUFAs, which directly influence the transcriptional activity of genes encoding these receptors (25).

In the alloxan-induced diabetic mice treated with ISS (diabetic control), histological analysis showed disruption of the liver cords and sinusoidal dilation, both of which are associated with obstruction of venous outflow. These results are in agreement with those reported by Samadder et al. (27) and Saadoun et al. (28). Furthermore, sinusoidal dilatation is also considered an important marker of inflammation and cellular damage in certain diseases such as hypertension, tumors, and diabetes (29,30). Our results showed that animals treated with the protein fraction of *U. dermestoides* or glibenclamide exhibited liver fibrotic regeneration, which should be confirmed in further studies.

Excessive protein intake can cause an overload for the kidneys and liver, both of which are responsible for removing waste substances (ammonia, urea, and uric acid) generated by excessive protein intake (31). In addition, excess protein disrupts liver tissue as the production of plasma proteins is reduced while transaminase levels are elevated. This may result in hepatotoxicity, inflammation and chronic liver damage, and could eventually lead to hepatic steatosis, fibrosis, cirrhosis or non-alcoholic fatty liver disease (32).

The fat fraction from *U. dermestoides* ameliorated the changes in liver histology induced by alloxan, with reduced sinusoidal dilatation and improved tissue organization observed in this group. This fat may play an important role in cell permeability, improving communication between cells and transport of proteins or receptors embedded in the membrane of liver cells (33). Like the lipid fraction, the protein fraction also exhibited a hypoglycemic effect, although mice that consumed the protein fraction also showed increased liver transaminases as a sign of liver damage. This finding is consistent with the liver damage observed in the micrographs. In contrast, the fat of *U. dermestoides* improved the hepatic architecture and promoted expression of *PPARγ* and *GLUT-4*, which might partly explain the positive effects of ingestion of *U. dermestoides* on glycemia in diabetic subjects.

Interestingly, in the pancreas, the fat fraction from *U. dermestoides* also augmented the number of islets, the cell density and regenerated the altered pancreatic architecture induced by alloxan. In the present investigation, the vasodilatation observed in the pancreas of mice in the group treated with this fat could be due to a pancreatic response to increase blood flow to the cells in order to obtain more nutrients. The higher cell density could lead to the activation of factors involved in vein dilation to satisfy the nutritional needs of the cells (34). In conjunction, these results may be indicative of islet regeneration. However, it is necessary to measure differentiation markers in future studies to clarify the nature of the new cells generated in the islets and to determine their origin.

Recent *in vivo* studies have shown that at least two transcription factors, Pax4 and Arx, can mediate transdifferentiation from α-cells to β-cells. It has been demonstrated that α-cells can continuously regenerate and convert into β-cells through overexpression of the transcription factor Pax4, which represses the master regulatory transcription factor Arx of α-cells, thereby stimulating the conversion of α-cells into β-cells (35). In addition, some compounds including γ-aminobutyric acid (GABA) and artemisinin antimalarial drugs can act as inducers of this cellular conversion (36). However, whether this process of cellular trans-differentiation in islets of Langerhans can be affected by the fat from *U. dermestoides* should be determined in further studies.

In relation to chitin, the main component of the exoskeleton of crustaceans and insects, its use is generally associated with beneficial effects (37). Our study showed that a dose of 16 mg·kg^-1^·day^-1^ of chitin extracted from *U. dermestoides* induced 100% lethality after 15 days of treatment. Its ingestion has been associated with inflammatory and allergic processes, which may cause death in diabetic subjects (38). Nevertheless, further toxicological studies with chitin isolated from *U. dermestoides* are mandatory. Other compounds of *U. dermestoides*, such as the benzoquinone volatile compounds isolated from the defensive secretions of *U. dermestoides*, have also been reported to cause cytotoxicity and genotoxicity in cultured cells (7,9), whereas phenolic compounds exhibited a free radical scavenging activity and antiproliferative effect in human lymphocytes (39,40).

In conclusion, the fat fraction from *U. dermestoides* produced a hypoglycemic effect in normal and diabetic mice, with evidence of regeneration of pancreatic islets, a liver-protective effect, and insulin-sensitizing properties. In addition, while other studies have associated the lipids from *U. dermestoides* with anti-irritant and central nervous system depressing effects (14,21), this is the first study to support the beneficial effects of *U. dermestoides* fat in diabetes treatment.
